# Investigation of Involvement between Specific Brain Regions and Evaluation Criteria Elements in a Two-Selections Task

**DOI:** 10.1155/2022/3999223

**Published:** 2022-12-20

**Authors:** Keita Mitani, Yukinobu Hoshino

**Affiliations:** ^1^Graduate School of System Engineering, Kochi University of Technology, Kochi, Japan; ^2^School of Systems Engineering, Kochi University of Technology, Kochi, Japan

## Abstract

It is essential to understand the neural mechanisms underlying human decision-making. Several studies using traditional analysis have attempted to explain the neural mechanisms associated with decision-making based on abstract rewards. However, brain-decoding research that utilizes the multivoxel pattern analysis (MVPA) method, especially research focusing on decision-making, remains limited. In brain analysis, decoding strategies for multivoxels are required for various decision-making evaluation criteria. This is because in daily life, the human decision-making process makes use of many evaluation criteria. In the present study, we investigated the representation of evaluation criterion categories in a decision-making process using functional magnetic resonance imaging and MVPA. Participants performed a decision-making task that involved choosing a smartphone by referring to four types of evaluation criteria. The regions of interest (ROIs) were the ventromedial prefrontal cortex (vmPFC), nucleus accumbens (NAcc), and insula. Each combination of the four evaluation criteria was analyzed based on a binary classification using MVPA. From the binary classification accuracy obtained from MVPA, the regions that reflected differences in the evaluation criteria among the ROIs were evaluated. The results of the binary classification in the vmPFC and NAcc indicated that these regions can express evaluation criteria in decision-making processes.

## 1. Introduction

Human decision-making, both simple and complex, occurs hundreds to thousands of times per day. A few examples may include decisions such as “What to eat,” “Which clothes to wear,” and “How to solve a problem.” Among these scenarios, purchase decision-making is a familiar and frequently encountered decision. When buying a product, especially a more expensive one, people tend to compare information to aid in the decision-making process. In recent years, online shopping has given people the opportunity to purchase a greater variety of products. However, due to the large variety of product lineups, it is difficult to make the best decision that satisfies consumer requirements. Furthermore, consumers cannot check the actual products; they can only view the product information, images, and reviews displayed on the screen. Therefore, decision-making regarding the purchasing of products should be supported. An important step in this direction would be to elucidate brain representation to gain a better understanding of the human decision-making process.

The field of neuro-marketing, which explores the human decision-making process by objectively measuring brain reactions and taking advantage of brain science and psychophysics knowledge, is attracting increasing attention [1, 2]. The neuroscience field uses methods such as functional magnetic resonance imaging (fMRI), functional near-infrared spectroscopy, positron emission tomography, and electroencephalography to explore human decision-making processes [3, 4]. Several studies using traditional univariate analysis have attempted to elucidate the neural mechanisms associated with decision-making based on abstract rewards.

One area that has been repeatedly shown to be activated by diverse rewarding stimuli is the ventromedial prefrontal cortex (vmPFC). In several neuroimaging studies, this brain region is active for a variety of primary and abstract rewards, including sports cars [5], cola preferences [6], pleasant odors [7], wine prices [8], facial attractiveness [9], and money [10]. The results of these past studies support the idea that the vmPFC is involved in converting the values of diverse stimuli into a common measure for a behavioral choice. Other findings have suggested that product preference activates the nucleus accumbens (NAcc). Knutson et al. [11] reported that preference elicits NAcc activation before a purchasing decision, whereas excessive prices can elicit insula activation and mesial prefrontal cortex (MPFC) deactivation.

Multivoxel pattern analysis (MVPA) has been attracting increasing attention, as shown in recent fMRI studies to elucidate brain activity patterns [12–14]. MVPA considers external stimuli, motion state, and mental content to be encoded in brain activity patterns. MVPA can save and distinguish spatial response patterns lost by averaging the responses across voxels in the region of interest (ROI), as in univariate analysis. When detecting the presence of a specific cognitive condition in the brain, the main advantage of MVPA is its increased sensitivity. The conventional fMRI analysis attempts to find voxels that exhibit statistically significant responses to experimental conditions. To increase the sensitivity to certain conditions, these methods spatially average the voxels that respond significantly to those conditions. Although this technique reduces noise, it also reduces the signal in two important ways. First, a voxel with a nonsignificant response to a particular condition can carry some information about the presence/absence of that state. Second, spatial averaging can blur the spatial pattern that distinguishes experimental conditions. As with conventional methods, the MVPA approach also attempts to increase sensitivity by looking at the contributions of multiple voxels. However, to avoid the signal loss problem mentioned above, MVPA does not routinely involve a uniform spatial average of the voxel response. Instead, it uses a weighted average of responses that treats each voxel as a separate source of information about the participant's cognitive state. This technique optimizes these weights and aggregates this information among voxels and finds ways to guide participants more accurately in terms of what they are thinking [15]. With this method, by analyzing a pattern composed of multiple voxels, it was possible to explore the brain expression of detailed information that could not be examined by conventional fMRI analysis. The traditional univariate approach focuses on the activity changes of each voxel. By contrast, MVPA extracts information from many brain locations (voxels) at the same time to examine the spatial brain activation pattern. MVPA is often used for neural decoding. Neural decoding is a technique to estimate stimuli, behaviors, and cognitive states. Several neural decoding studies are being conducted to reconstruct visual information, cognitive judgments, and emotions [16–18]. Machine learning methods such as the support vector machine and neural network are important for this approach. Neural decoding is realized by learning brain activity patterns, which are multidimensional variables, using a machine learning algorithm and outputting prediction values from new brain activity patterns using the learned model. There are many studies on biometric data classification using machine learning other than neural decoding. In the medical field, research is being conducted to detect lesions and classify benign/malignant tumors, and machine learning methods are used [19, 20].

A relatively large amount of the existing research using MVPA focuses on decision-making because it is important to understand which brain regions are involved in the various metrics used during decision-making in daily life. Given this background, the present study aimed to estimate user choice in decision-making based on brain activity. To achieve this aim, we experimented to verify the brain regions involved in the evaluation criteria in decision-making processes and investigated the representation of their categories using fMRI and MVPA. Assuming a situation involving the purchase of smartphones through online shopping, participants picked one from a choice of two products with information for a single evaluation criterion. This task involved four types of evaluation criteria for decision-making. We focused on the voxel pattern in ROIs based on differences in the type of evaluation criteria. To our knowledge, brain regions reflecting the type of evaluation criteria used in decision-making have not been reported.

In our previous experiments, we evaluated the statistical significance for the whole brain and found that the brain regions in which each evaluation criterion was specifically involved were not observed for all evaluation criteria. Moreover, no significant difference in brain activity was observed for each evaluation criterion in the ROI [21]. We hypothesized that the type of evaluation criteria is specific to or commonly involved in certain brain regions. This analysis focused on the vmPFC, insula, and NAcc, which are considered to be involved in decision-making and were used as ROIs [22–26]. We hypothesized that there would be a difference in the activation patterns of the vmPFC, insula, and NAcc depending on the type of evaluation criteria. To examine this hypothesis, a decision-making task regarding each evaluation criterion was performed. The differences in brain activation patterns due to the differences in evaluation criteria in these ROIs were then examined by comparing each combination of evaluation criteria. For the analysis, we used MVPA, which has been established as an effective method for identifying and classifying brain activity patterns. Based on different evaluation criteria, the voxel patterns from all associated regions were examined using MVPA. Each combination of the four evaluation criteria was analyzed after binary classification by MVPA. Finally, based on the binary classification accuracy obtained from MVPA, the regions among the ROIs that reflected differences in evaluation criteria were evaluated.

For many people, evaluation criteria such as price, color, and production date are important factors in making a better purchase. The results obtained in this experiment indicate that vmPFC and NAcc respond concerning the same endpoints when making a choice. Moreover, as shown in [8, 11, 22, 23, 25], the vmPFC and NAcc are sites involved in decision-making and execution. On the contrary, there are neuromarketing methods that take advantage of findings from brain science and apply them to marketing activities [27–29]. It is important to analyze the consumer psychology and behavioral principles indicated by brain science. The traditional methods of behavior analysis were questionnaires or interviews and thus could not elicit consumers' unconscious true intentions. However, the results of this research proved that the vmPFC and NAcc measurements can be used as a model to visualize and quantitatively evaluate consumers' unconscious psychology and preferences, which are difficult to verbalize.

## 2. Materials and Methods

The materials and methods used to in this article are described in the following sections.

### 2.1. Participants

Twenty-five participants (five females: two left-handed, mean age 20.60 years, standard deviation 1.26 years, and age range 19–22 years) participated in the fMRI experiment. One participant who did not complete the experiment was excluded. Therefore, 24 participants were finally included in the data analysis. This study was approved by the Research Ethics Review Committee of the Kochi University of Technology (approval no. 52-C3). All participants provided written informed consent before the experiment began.

### 2.2. Task and Stimuli

**Figure 1:**
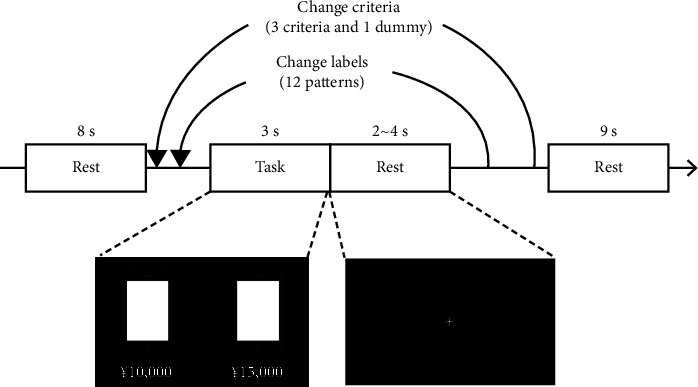
Experimental timeline.

All participants performed a decision-making task that involved choosing a smartphone by referring to each evaluation criterion.  Figure 1 shows the experimental timeline. This experiment was based on a block design. It consists of several discrete periods of on-off blocks, with the “on” representing a task condition and the “off” referring to a rest state or a different task condition.

In this task, participants viewed a screen, an example of which is illustrated in the lower part of Figure 1. The screen presented two identical smartphone images and the letters of each different combination of evaluation criteria as stimuli. This experimental design using these stimuli, which are illustrated in two identical images, each with different information, has also been adopted in other decision-making studies [30]. The participants pushed the left or right button to select the smartphone they wanted more.

The experimental conditions included the price, body color, and production year as the evaluation criteria. The body color evaluation was conveyed through textual information instead of an illustration. The reason for this was to avoid the possibility of a difference in willingness to purchase depending on the displayed color of the product [31]. These choices were selected as evaluation criteria because they can be evaluated easily regardless of the presence or absence of smartphone knowledge among the participants. In addition, a dummy variable (four squares as meaningless symbols) was set as the control condition. The brain activity at the time of the main task was considered to include three main effects: decision-making, visual recognition of the stimuli, and button-pressing at the time of selection. In addition, brain activity during dummy tasks is thought to reflect the effects of almost the same condition as the main task, except for decision-making. Brain activity regarding the difference between the main and dummy tasks is considered to represent only the effects of decision-making. Each criterion had four types of content.  Table 1 shows the list of evaluation criterion labels used in this experiment. Each participant was considered to have a different priority for each evaluation criterion. In this experiment, it was hypothesized that a specific brain-related region would refer to the impression for each evaluation criterion that included priority differences. The evaluation criteria appeared in a different order for each participant. Each participant performed two runs under the same conditions. Each run contained eight blocks of four separate tasks: the price choice, the color choice, the year choice, and the dummy choice. During each choice, a screen showed information for 3 seconds, followed by a rest period for 2–4 seconds. The screen presented two images of the smartphone, shown on the left and right of the screen. Different labels under the images represent each evaluation criterion. In the choice tasks, the participants decided on an object in their mind and then pushed the left or right button to select the object they had chosen. In the dummy tasks, the participants were required to push either button intuitively. The total time of one run was 306 seconds. Stimuli images and words were rear-projected onto a screen placed in the scanner bore using an LCD projector. The screen showed two identical smartphone images and two different labels as an evaluation criterion, as demonstrated in Figure 1.

### 2.3. MRI Acquisition and Data Preprocessing

Scanning was performed on a 3.0-tesla scanner (MAGNETOM Verio, Siemens Healthinners, Erlangen, Germany) using a 16-channel head coil at the Kochi University of Technology. Functional scans were acquired with a standard gradient-echo echo-planar imaging sequence to cover the whole brain (field of view = 192 mm^2^; repetition time = 3,000 ms; echo time = 30 ms; flip angle = 90°; slice thickness = 3.0 mm; voxel size = 3.0 mm^3^). Each run of the functional scans obtained 102 volumes over a total duration of 306 seconds. A high-resolution T1-weighted anatomical scan was acquired for each subject (1.0 mm^3^ resolution).

The first two scans (6 seconds) in each run were discarded to account for any instability with the fMRI scanner. SPM12 software (Wellcome Centre for Human Neuroimaging, London, UK) was used to process and analyze the functional data. Functional images were corrected for differences in slice acquisition time and motion. The data were then realigned and normalized to the Montreal Neurological Institute (MNI) standard brain model. The brain activation degrees were analyzed on the MNI coordinates.

### 2.4. fMRI Analyses

The following four conditions were modeled: price choice, color choice, year choice, and dummy choice. Common or specific brain regions were involved in each condition, and these regions were identified by creating contrasts. With the first level (single subject analysis), contrasts (price vs. dummy, color vs. dummy, and year vs. dummy) were created to identify brain regions, which were commonly activated for all contrasts. Price choice vs. the two other choices, color choice vs. the two other choices, and year choice vs. the two other choices were created to identify brain regions, which were specifically activated for each contrast. With the second level (group analysis), one sample *t* tests were performed to examine significant brain activation among the group during the contrasts mentioned. A statistical parametric map was generated using the price, color, and year vs. dummy choice contrasts. Clusters of voxels were corrected for multiple comparisons across the whole brain using family-wise error correction and a threshold of *p* values: *p* < 0.05 [32]. The statistical parametric maps were generated using color vs. (price and year) choice contrast. Clusters were defined using a height threshold of *p* < 0.001 uncorrected for multiple comparisons with a cluster size threshold of *k* = 171 voxels. In the contrasts of price vs. (color and year) and year vs. (price and color), no suprathreshold clusters were applied.

### 2.5. Multivoxel Pattern Analysis

The results of the fMRI analysis described in the previous section showed activation in the vmPFC during decision-making about each evaluation criterion. Brain activation patterns in the decision-making task were investigated using MVPA based on each evaluation criterion.

MVPA was performed using a support vector machine (SVM) with a linear kernel [33], as implemented in the Pattern Recognition for Neuroimaging Toolbox [26]. The pattern analyses were performed separately for each participant. The *β* value at decision-making based on each evaluation criterion obtained from the general linear model in the previous section was taken as the input value. There were 48 *β* belonging to four evaluation criteria, including the dummy, from two runs for each participant (24/run). A binary classification according to the four evaluation criteria was carried out, with each beta representing a single decision-making event based on an evaluation criterion. As the evaluation criterion for each *β* is already known, in MVPA, these beta values were labeled “price,” “color,” “year,” and “dummy.” The beta values, which were labeled as two types of evaluation criteria, were input to the SVM as training sets to generate a boundary of two classes. We examined into which class the sample data with either label were classified.

The analysis was performed using voxels in only the vmPFC, insula, or NAcc by masking using the PickAtlas toolbox. In this analysis, binary classification by SVM was performed to test whether every ROI could distinguish between the four evaluation criteria (e.g., “price or color” and “color or year”). WFU PickAtlas [34, 35] was used to create the three ROI masks for the vmPFC, insula, and NAcc. The mask for vmPFC selected the Brodmann areas 10, 14, 25, and 32 as defined by Finger [36]. Data were cross-validated using the leave one block out method, with two sets of 24 data points from each participant. Only one sample data point was extracted from all the data as a test set. The remaining data points were used as a training set. The verification was repeated using all the sample data as a test set. The number of correct answers among the 48 data points by each participant (e.g., when price data were classified as price) was obtained from the SVM. Next, the average correct answer rate for each binary classification obtained from all participants was calculated. The average correct answer rates were evaluated to reveal whether the brain activity pattern of each ROI expressed the evaluation criteria for decision-making.

## 3. Results

We performed a whole brain analysis to identify regions that had significant activation for each choice.  Table 2 shows the key brain activation patterns when choosing each pair of alternatives. Regarding price vs. dummy, the left occipital gyrus and right calcarine showed significant activity differences. Regarding color vs. dummy, the left fusiform gyrus, bilateral occipital gyri, right lingual gyrus, bilateral superior frontal gyri, left insula, right cerebellum, left triangular part of the inferior frontal gyrus, right calcarine, right middle frontal gyrus, and right angular gyrus showed significant activity differences. Regarding year vs. dummy, the bilateral occipital gyri, right inferior temporal gyrus, and right cerebellum showed significant activity differences.

**Figure 2:**
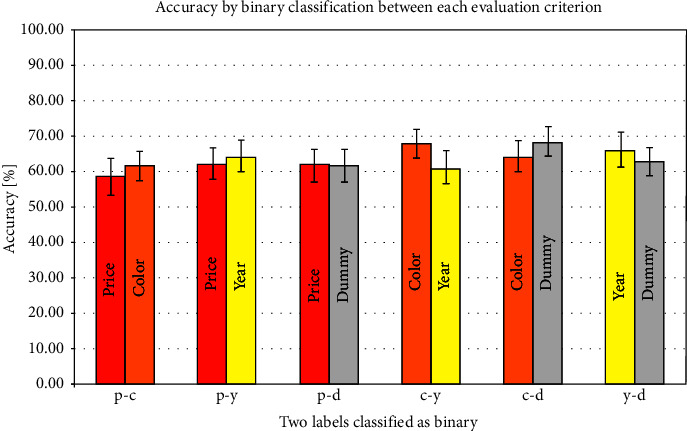
Correct answer rate in the vmPFC.

**Figure 3:**
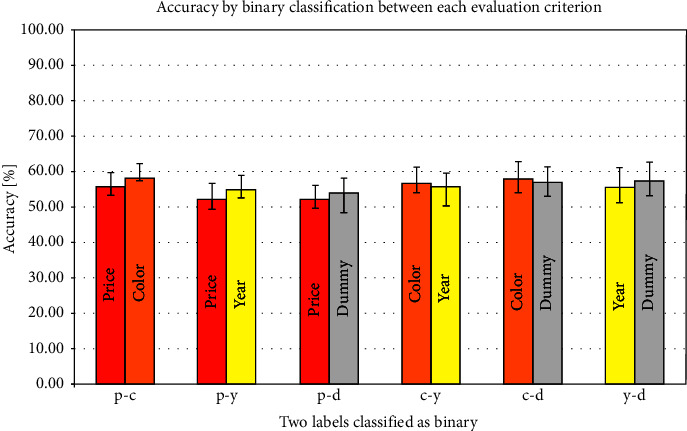
Correct answer rate in the insula.

**Figure 4:**
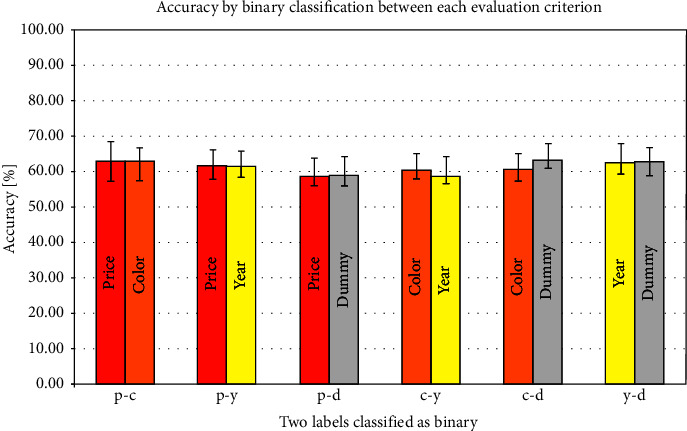
Correct answer rate in the NAcc.

The precision results for the binary classification in vmPFC, insula, and NAcc are shown in Figures 2–4 and Table 3. In these figures, the color bars represent the average value of all 24 participants, and the error bars represent standard errors. The *x*-axis shows a combination of each binary classification (e.g., p-c shows the result of binary classification by price and color). In the precision using the vmPFC as an ROI, the highest average accuracy was 68.40% in the dummy-color binary classification, and the lowest was 58.51% in the price-color binary classification. Regarding the result of the insula as ROI, the highest average accuracy was 58.33% in the color-price binary classification and the lowest was 52.08% in the price-dummy binary classification. Regarding the result of the NAcc as an ROI, the highest average accuracy was 63.37% in the dummy-color binary classification and the lowest was 58.51% in the price-dummy binary classification.

## 4. Discussion

The present study investigated the neural substrates associated with assessments of different criteria in decision-making. To this end, the study participants were presented with a pair of alternatives belonging to a single evaluation criterion and asked which product they wanted to choose based on the evaluation criteria. We evaluated whether decision-making based on different evaluation criteria could be discriminated based on the spatial activity pattern in different brain regions.

Considering the influence of the context of the vmPFC, insula, and NAcc activities observed in past studies, the ROIs of those areas were used. The vmPFC is active for a variety of primary and abstract rewards in several neuroimaging studies [22–25]. These findings suggest that the vmPFC plays several roles in the representation of complex choices, which suggests that the activated region differs depending on the evaluation criteria when based on an alternative value or preference by each individual. The insula has shown the possibility of triggering activation for the price during a purchasing decision and the NAcc of inducing activation for individual preferences [26]. These findings suggest that the brain frames a preference as a potential benefit and price as a potential cost, thereby lending credence to the notion that consumer purchasing reflects an anticipatory combination of preference and price considerations.

Although few studies have investigated the influence of differences in evaluation criteria on the brain, numerous studies have examined the influence of preferences and pleasure/discomfort when participants evaluate two or more stimuli as a decision-making task on the brain. These experimental designs are roughly divided into two types. The first is a design that presents a single stimulus in order, a design that the subject subsequently carries out in a single evaluation. The second presents a pair of stimuli at the same time, and the subjects select what they prefer more. In the present experiment, the latter design was adopted, in which the subjects evaluated a pair of alternatives belonging to a certain evaluation standard through comparisons. Numerous studies on decision-making have presented a pair of images or character strings and performed evaluations, while others have used an experimental design that presents the same image and character strings of different contents, such as the present experimental design. Some studies have also reported results regarding activity in the vmPFC, insula, and NAcc [22, 23, 25].

As for the average accuracy of binary classification by MVPA, the present results using vmPFC, insula, and NAcc as ROIs exceeded the chance level. In the insula, even though the chance level exceeded the average precision, the standard error of the binary classification accuracy of several combinations was lower than the chance level. The average classification accuracy in the insula was lower than those in the vmPFC and NAcc for all combinations of evaluation criteria. The average classification accuracy in the NAcc was about 60%, and no difference in classification accuracy was seen for any evaluation criterion. On the contrary, vmPFC showed a difference in the binary classification accuracy for each combination of evaluation criteria. In the binary classification of price and year, the year was 2.26% more accurate. The color was 2.95% more accurate than the price and 6.60% more accurate than the year. Although none of the differences in average accuracy were significant, classification in the vmPFC showed the possibility of being most classifiable when color was used as the evaluation criterion. These results suggest that brain regions involved in the decision and the preference, such as vmPFC and NAcc, represent differences in brain activity patterns in comparative decision-making.

Several studies applying MVPA to brain activity during decision-making have reported the following results: Jai et al. conducted an evaluation and bidding task for apparel products under three visual presentation conditions (static picture, zooming, or model rotation videos) and analyzed the brain patterns in “a buy decision” and “a not buy decision” [38]. According to the whole-brain classification analysis, the classifier accuracy rates were 95% in the zooming and rotation conditions, while the static condition had a 75%. By the searchlight classification, there were some ROIs exclusively referred by certain presentations. These results suggest that the brain regions to which various information corresponds exhibit characteristic activity patterns and that appropriate recognition of these patterns enables classification. On the contrary, Kim et al. investigated the relationship between purchase intention and perceived garment fit when purchasing decision-making [39]. They analyzed the brain activity during the task including the phase of evaluating the fit of the model wearing clothes and the phase of purchasing consideration with the price displayed on the clothes. As a result of the MVPA for whole brain searchlight, the classification accuracies were more than 50% of the chance level at 11 brain regions. Among them, the superior parietal lobule exceeded 80% with the highest accuracy. One of the reasons for this accuracy higher than our results may be that their tasks included multiple evaluation criteria such as garment fit and price. Our results showed that decision-making based on individual criteria is represented in brain patterns, but we believe that decision-making based on multiple criteria may represent more characteristic brain patterns.

This study focused on customer preferences based on individual evaluation criteria. Although the results of the classifier did not show activity patterns in specific brain regions that depended on the evaluation criteria, they indicated the possibility that preference-based decision-making could be represented in brain patterns. It is necessary to process various factors such as reward, risk, and strategy in decision-making. In particular, regarding purchasing decisions, it is said that the factor of individual preferences produces better decisions [40–43]. The development of this research is expected to help elucidate the neural basis of decision-making and to contribute to selection, online shopping, or marketing strategies.

## 5. Conclusions

This study aimed to estimate user choice in decision-making based on brain activity. The experiment focused on evaluation criteria, which motivate decision-making, and the verification of brain regions involved in these evaluation criteria. To achieve our purpose, we investigated the representation of evaluation criterion categories in decision-making using fMRI and MVPA. Price, color, and year were used as the evaluation criteria. We focused on the vmPFC, NAcc, and insula as ROIs. Each combination of the four evaluation criteria was analyzed into a binary classification by MVPA. From the binary classification accuracy obtained from MVPA, we evaluated the regions that reflected differences in evaluation criteria among the ROIs. From the results of the binary classification by MVPA, the vmPFC and NAcc showed that these regions were capable of expressing the influence of the evaluation criteria during decision-making.

In this research, we applied classification analysis from brain activity by adopting price, color, and year as evaluation criteria because they are often involved in purchasing decisions for various products. In actual purchase decision-making, consumers also refer to various evaluation criteria. In a future study, it will be necessary to investigate brain activity patterns expressed by evaluation criteria other than those adopted in the present study and to verify whether they can be classified similarly. The present analysis involved only binary classification by two classes. As a future challenge, the accuracy of multiclass classification will need to be verified.

## Figures and Tables

**Table 1:** List of evaluation criterion contents.

Evaluation criteria	Labels			
Price	¥10,000	¥15,000	¥20,000	¥25,000
Color	Black	White	Red	Blue
Year	2004	2008	2012	2016
Dummy	□□□□			

Note: ¥ sign means Japanese yen.

**Table 2:** Common significant brain activation for each label.

Region label	Cluster size	*T*-statistic	MNI coordinates		
			*x*	*y*	*z*
Price vs. dummy					
Occipital Mid L	482	9.35	−22	−96	0
Calcarine R	363	8.80	24	−92	2

Color vs. dummy					
Fusiform L	484	8.94	−40	−54	−10
Occipital Inf R	59	8.41	36	−84	−12
Lingual R	91	8.01	12	−90	−4
Frontal Sup Medial R	15	7.60	6	24	42
Occipital Mid R	31	7.38	32	−66	26
Insula L	16	7.13	−30	18	−4
Cerebellum 9 R	5	6.90	6	−56	−40
Frontal Inf Tri L	4	6.83	−38	38	10
Calcarine R	7	6.83	6	−62	12
Occipital Sup R	8	6.81	30	−72	46
Frontal Sup 2 L	1	6.78	−12	50	38
Frontal Mid 2 R	1	6.75	36	52	−2
Occipital Mid L	24	6.67	−30	−78	24
Frontal Mid 2 R	1	6.53	30	54	4
Occipital Mid L	1	6.50	−28	−80	18
Angular R	1	6.44	34	−70	46

Year vs. dummy					
Occipital Inf L	464	10.26	−38	−82	−10
Occipital Inf R	527	9.80	22	−92	−4
Occipital Mid L	23	7.69	−30	−80	24
Temporal Inf R	19	6.95	50	−64	−12
Cerebellum 6 R	1	6.45	10	−74	−18

Note: region labels were named based on the automated anatomical labeling template [37], which is a digital human brain Atlas with a labeled volume. The labels indicate macroscopic brain structures. Cluster size is reported in voxels. The *T*-statistic value is the total average, which was calculated for each voxel from MRI data acquired for each subject and divided by the standard deviation of all subjects.

**Table 3:** Average accuracies and standard errors by binary classification between each evaluation criterion.

Regions of interest	Combinations of evaluation criteria											
	p-c	c-p	p-y	y-p	p-d	d-p	c-y	y-c	c-d	d-c	y-d	d-y
vmPFC												
Average accuracy (%)	58.5	61.5	62.2	64.4	61.5	61.5	67.7	61.1	64.2	68.4	66.0	62.7
± standard error	5.3	4.0	4.4	4.3	4.7	4.8	4.2	4.7	4.4	4.1	5.0	4.1

Insula												
Average accuracy (%)	56.0	58.3	52.8	54.7	52.1	54.3	57.1	55.9	57.5	57.3	55.9	57.6
± standard error	4.0	4.5	4.3	4.4	4.5	4.5	4.3	4.4	4.8	4.7	5.4	5.4

NAcc												
Average accuracy (%)	63.0	63.0	61.6	61.5	58.5	59.0	60.4	59.2	60.6	63.4	62.7	63.0
± standard error	4.4	4.0	4.6	4.4	5.3	5.4	4.8	5.0	4.5	4.6	5.2	4.9

## Data Availability

The data used to support the findings of the study can be obtained from the corresponding author upon request.

## References

[1] Ariely D., Berns G. S. (2010). Neuromarketing: the hope and hype of neuroimaging in business. *Nature Reviews Neuroscience*.

[2] Plassmann H., O’Doherty J., Rangel A. (2007). Orbitofrontal cortex encodes willingness to pay in everyday economic transactions. *Journal of Neuroscience*.

[3] Kopton I. M., Kenning P. (2014). Near-infrared spectroscopy (NIRS) as a new tool for neuroeconomic research. *Frontiers in Human Neuroscience*.

[4] Pisauro M. A., Fouragnan E., Retzler C., Philiastides M. G. (2017). Neural correlates of evidence accumulation during value-based decisions revealed via simultaneous EEG-fMRI. *Nature Communications*.

[5] Erk S., Spitzer M., Wunderlich A. P., Galley L., Walter H. (dec 2002). Cultural objects modulate reward circuitry. *NeuroReport*.

[6] McClure S. M., Li J., Tomlin D., Cypert K. S., Montague L. M., Montague P. (2004). Neural correlates of behavioral preference for culturally familiar drinks. *Neuron*.

[7] Rolls E. T., Kringelbach M. L., De Araujo I. E. T. (2003). Different representations of pleasant and unpleasant odours in the human brain. *European Journal of Neuroscience*.

[8] Plassmann H., O’Doherty J., Shiv B., Rangel A. (2008). Marketing actions can modulate neural representations of experienced pleasantness. *Proceedings of the National Academy of Sciences*.

[9] O’Doherty J., Winston J., Critchley H., Perrett D., Burt D., Dolan R. (jan 2003). Beauty in a smile: the role of medial orbitofrontal cortex in facial attractiveness. *Neuropsychologia*.

[10] Knutson B., Fong G. W., Bennett S. M., Adams C. M., Hommer D. (2003). A region of mesial prefrontal cortex tracks monetarily rewarding outcomes: characterization with rapid event-related fMRI. *NeuroImage*.

[11] Knutson B., Rick S., Wimmer G. E., Prelec D., Loewenstein G. (2007). Neural predictors of purchases. *Neuron*.

[12] Kamitani Y., Tong F. (2005). Decoding the visual and subjective contents of the human brain. *Nature Neuroscience*.

[13] Norman K. A., Polyn S. M., Detre G. J., Haxby J. V. (2006). Beyond mind-reading: multi-voxel pattern analysis of fMRI data. *Trends in Cognitive Sciences*.

[14] Chikazoe J., Lee D. H., Kriegeskorte N., Anderson A. K. (2014). Population coding of affect across stimuli, modalities and individuals. *Nature Neuroscience*.

[15] Meyer K., Kaplan J. T. (2011). Cross-modal multivariate pattern analysis. *Journal of Visualized Experiments: Journal of Visualized Experiments*.

[16] Katsikopoulos K., Canellas M. (2022). Decoding human behavior with big data? critical, constructive input from the decision sciences. *AI Magazine*.

[17] Yau Y., Dadar M., Taylor M. (2020). Neural correlates of evidence and urgency during human perceptual decision-making in dynamically changing conditions. *Cerebral Cortex*.

[18] Liu Z., Liu S., Li S. (2022). Dissociating value-based neurocomputation from subsequent selection-related activations in human decision-making. *Cerebral Cortex*.

[19] Chowdhary C. L., Mittal M., Kumaresan P., Pattanaik P. A., Marszalek Z. (2020). An efficient segmentation and classification system in medical images using intuitionist possibilistic fuzzy c-mean clustering and fuzzy svm algorithm. *Sensors*.

[20] Rizwan M., Shabbir A., Javed A. R., Shabbir M., Baker T., Al-Jumeily Obe D. (2022). Brain tumor and glioma grade classification using Gaussian convolutional neural network. *IEEE Access*.

[21] Keita M., Hoshino Y. (2018). Verification for commonality or specificity of brain representations related to the different evaluation criteria. *International Journal of Innovative Computing Information and Control*.

[22] Dhar R., Wertenbroch K. (2000). Consumer choice between hedonic and utilitarian goods. *Journal of Marketing Research*.

[23] Hoch S. J., Loewenstein G. F. (mar 1991). Time-inconsistent preferences and consumer self-control. *Journal of Consumer Research*.

[24] Mellers B. A., Chang S.-J., Birnbaum M. H., Ordonez L. D. (1992). Preferences, prices, and ratings in risky decision making. *Journal of Experimental Psychology: Human Perception and Performance*.

[25] Lindbladh E., Lyttkens C. H. (aug 2002). Habit versus choice: the process of decision-making in health-related behaviour. *Social Science & Medicine*.

[26] Oosterhof N. N., Connolly A. C., Haxby J. V. (jul 2016). CoSMoMVPA: multi-modal multivariate pattern analysis of neuroimaging data in matlab/GNU octave. *Frontiers in Neuroinformatics*.

[27] Nilashi M., Samad S., Ahmadi N. (2020). Neuromarketing: a review of research and implications for marketing. *Journal of Soft Computing and Decision Support Systems*.

[28] Mansor A. A., Mohd Isa S. (2020). Fundamentals of neuromarketing: what is it all about?. *Neuroscience Research Notes*.

[29] Alsharif A. H., Salleh N. Z., Baharun R. O., Yusoff M. E. (2021). Consumer behaviour through neuromarketing approach. *Journal of Contemporary Issues in Business and Government*.

[30] Cherry J. B. C., Bruce J. M., Lusk J. L., Crespi J. M., Lim S. L., Bruce A. S. (2015). Neurofunctional correlates of ethical, food-related decision-making. *PLoS One*.

[31] Garber L. L., Burke R. R., Morgan Jones J. (2000). *The Role of Packaging Color on Consumer purchase Consideration and Choice*.

[32] Friston K. J., Holmes A. P., Worsley K. J., Poline J. P., Frith C. D., Frackowiak R. S. J. (1994). Statistical parametric maps in functional imaging: a general linear approach. *Human Brain Mapping*.

[33] Chang C.-C., Lin C.-J. (2011). Libsvm: a library for support vector machines. *ACM transactions on intelligent systems and technology (TIST)*.

[34] Maldjian J. A., Laurienti P. J., Kraft R. A., Burdette J. H. (2003). An automated method for neuroanatomic and cytoarchitectonic atlas-based interrogation of fMRI data sets. *NeuroImage*.

[35] Maldjian J. A., Laurienti P. J., Burdette J. H. (2004). Precentral gyrus discrepancy in electronic versions of the Talairach atlas. *NeuroImage*.

[36] Finger E. C., Marsh A. A., Mitchell D. G. (2008). Abnormal ventromedial prefrontal cortex function in children with psychopathic traits during reversal learning. *Archives of General Psychiatry*.

[37] Tzourio-Mazoyer N., Landeau B., Papathanassiou D. (2002). Automated anatomical labeling of activations in SPM using a macroscopic anatomical parcellation of the MNI MRI single-subject brain. *NeuroImage*.

[38] Jai T. M. C., Fang D., Bao F. S., James R. N., Chen T., Cai W. (2021). Seeing it is like touching it: unraveling the effective product presentations on online apparel purchase decisions and brain activity (an fmri study). *Journal of Interactive Marketing*.

[39] Kim H. E., Kwon J. H., Kim J. J. (2021). Neural correlates of garment fit and purchase intention in the consumer decision-making process and the influence of product presentation. *Frontiers in Neuroscience*.

[40] Simonson I., Nowlis S. M. (2000). The role of explanations and need for uniqueness in consumer decision making: unconventional choices based on reasons. *Journal of Consumer Research*.

[41] Dhar R. (1997). Consumer preference for a no-choice option. *Journal of Consumer Research*.

[42] Ariely D. (2000). Controlling the information flow: effects on consumers’ decision making and preferences. *Journal of Consumer Research*.

[43] Slovic P. (1995). The construction of preference. *American Psychologist*.

